# Back to life: Biological parenthood of young adult acute leukaemia survivors

**DOI:** 10.1111/bjh.70107

**Published:** 2025-08-26

**Authors:** Katharina Egger‐Heidrich, Katja Sockel, Johannes Schetelig

**Affiliations:** ^1^ Department of Internal Medicine I University Hospital, TU Dresden Dresden Germany; ^2^ DKMS Group Tübingen, Dresden Germany

**Keywords:** acute leukaemia, allogeneic haematopoietic stem cell transplantation, chemotherapy, fertility, survivorship

## Abstract

Commentary on: Nielsen et al. Reproductive patterns and birth rates in acute leukemia survivors: A Danish population‐based cohort study. Br J Haematol 2025; 207:1435–1444.

In their paper, Nielsen et al. present data from a Danish population study on birth rates among acute leukaemia survivors aged 18–45 years at the time of leukaemia diagnosis.^1^ While acute leukaemia survivors had a lower birth rate and higher use of assisted reproduction technology (ART), adverse pregnancy outcomes were not observed more often compared to matched leukaemia‐free counterparts. Several aspects and results of this analysis are outstanding.

First, the authors analysed data from the Danish National Acute Leukemia Registry, the Danish National Pathology Register, the Danish Patient Registry, the Danish Civil Registration System, the Danish Medical Birth Register and the In Vitro Fertilization (IVF) Register. Mapping of individuals with acute leukaemia and leukaemia‐free comparators across these registers allowed for an extraordinary complete dataset. Live birth rate ratios are based on 106 live births, including 71 first live births of 401 leukaemia survivors of whom 158 patients had received alloHCT. Their data were compared to data from 4010 control individuals from the general Danish population without acute leukaemia, who were matched for sex, age and parenthood status, for whom 1,564 live births, including 986 first live births, had been reported. It is a positive surprise that first birth incidence rate ratios among leukaemia survivors were only 25% lower for female patients (IRR 0.75, 95% CI 0.52–1.08) and 21% lower for male patients (IRR 0.75, 95% CI 0.58–1.09) compared to leukaemia‐free individuals respectively. Similarly, among patients who had received alloHCT, nominally lower birth rates were seen in female (IRR 0.46, 95% CI 0.19–1.12) and male patients (IRR 0.73, 95% CI 0.38–1.37) (Figure 1). Interestingly, the reduction of birth rate for the female Danish 3‐year relapse‐free acute leukaemia survivors with alloHCT was less pronounced than the birth rates of all female transplant survivors from a German cohort study.^2^

**FIGURE 1:**
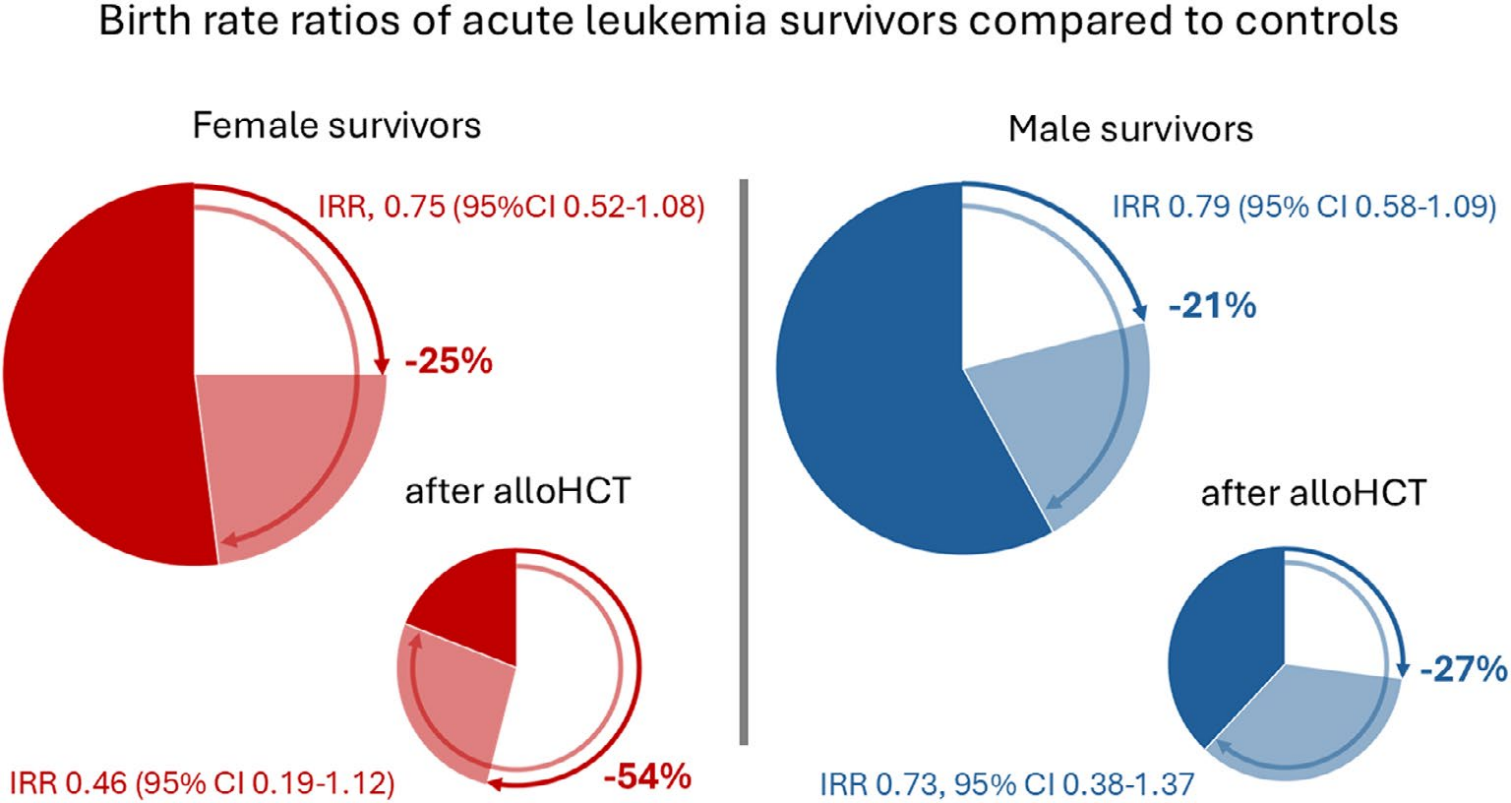
Birth rate ratios of acute leukaemia survivors compared to controls. The pie charts show first live birth rate ratios of 3‐year relapse‐free acute leukaemia survivors aged 18–45 years at the time of diagnosis. For patients after allogeneic haematopoietic cell transplantation (alloHCT), all live birth rate ratios are displayed. The estimates are based on 106 live births, including 71 first live births of 401 leukaemia survivors of whom 158 patients had received alloHCT compared to 1564 live births, including 986 first live births, of 4010 controls. Controls from the general Danish population without acute leukaemia diagnosis were matched for sex, age and parenthood status. Full arrows and white pie segments visualize the reduction of birth rates for female and male survivors. Transparent pie segments and arrows visualize the lower margin of the 95% confidence interval of the IRR. alloHCT, allogeneic haematopoietic cell transplantation; IRR, live birth incidence rate ratio.

Second, the resulting dataset is uniquely comprehensive, for example, on marital status, use of ART and birth outcomes. Notably, 50% of female leukaemia survivors were married versus 45% of female matched controls and 43% of male leukaemia survivors versus 38% of male matched controls, suggesting comparable social functioning and well‐being of most leukaemia survivors in this domain.^3^ The promising birth rates were achieved with a higher use of ART among leukaemia survivors compared to their matched comparators (females: 25.5% versus 14.1%; males: 55.9% versus 12.2%). But once again, it nourishes optimism that 74.5% and 44.1% of live births among female and male leukaemia survivors resulted from natural conception respectively. No children were stillborn during the study period, and there was no difference in the number of pre‐ or post‐mature deliveries between leukaemia survivors and controls.

While this study adds another important piece of information on fertility of leukaemia survivors, more research is needed. Most important, fertility preservation should be investigated in the context of specific treatment courses in order to better understand and select fertility preserving treatment options. This is especially important with respect to conditioning prior to cellular therapies. In this context, carefully designed prospective observational studies to investigate hormonal recovery as a surrogate end‐point might accelerate knowledge on fertility‐preserving treatment options.

Parenthood is an important topic for all adolescents and young adults (AYA) aged 15–39 years. However, for those AYA diagnosed with acute leukaemia, the diagnosis and its intensive treatment depict a massive turning point in life. Beyond achieving leukaemia‐free survival, returning to a largely normal life should be a key treatment goal. For many of those affected, the desire to have their own biological children is a central issue in life. Unfulfilled desire to have children can have a lasting impact on the quality of life and may also affect partnership and social functioning. Therefore, fertility preservation measures, whenever feasible, are of greatest importance. Guidelines on this topic have been published for female and male AYA and adults by the American Society of Clinical Oncology and International Guideline Harmonization Group for late effects of childhood cancer in collaboration with the PanCare Consortium.^4^, ^5^, ^6^

Fertility counselling is highly recommended for young leukaemia long‐term survivors with an unfulfilled desire of parenthood. The data from Denmark suggest that this might be needed less often than apprehended. Given the rate of spontaneous pregnancies, leukaemia survivors in reproductive age should be at the same time made aware of the possibility of an unwanted pregnancy. These aspects should be part of tailored risk‐adapted long‐term follow‐up care, for example, in specialized survivorship clinics.

## AUTHOR CONTRIBUTIONS

All authors contributed to a similar extent to writing and review of the manuscript.

## Data Availability

Data sharing is not applicable to this article as no new data were created or analyzed in this study.
